# Interactions Between Paracetamol and Hypromellose in the Solid State

**DOI:** 10.3389/fphar.2019.00014

**Published:** 2019-01-24

**Authors:** Edyta Leyk, Marek Wesolowski

**Affiliations:** Department of Analytical Chemistry, Medical University of Gdansk, Gdansk, Poland

**Keywords:** paracetamol, hypromellose, interactions, phase diagram, solubility with polymer, miscibility with polymer, glass transition in mixture

## Abstract

Hydroxypropyl methylcellulose (hypromellose) is a widely known excipient commonly used in the preparation of drug formulations. It can interact with some active pharmaceutical ingredients (APIs), thereby contributing to a reduction in crystallinity, serve as a solvent for API or form stable dispersion with no tendency to aggregation. The aim of the present study was to investigate the effect of hypromellose on the solubility, miscibility and amorphization of paracetamol in mixture with this polymer. Homogenized mixtures of paracetamol with hypromellose were studied using differential scanning calorimetry (DSC), hot-stage microscopy (HSM), Fourier transform infrared (FT-IR) and Raman methods to obtain a deeper insight into the interactions between ingredients in solid state including phase diagram construction for crystalline API and amorphous polymer. A DSC study revealed potential interaction between ingredients resulting in reduced paracetamol crystallinity. This was proved using heating-cooling-heating test to confirm paracetamol amorphization. FT-IR and Raman investigations excluded chemical reaction and hydrogen bonding between ingredients. The phase diagram developed facilitates predictions on the solubility of API in polymer, on the mutual miscibility of ingredients and on the temperature of mixture glass transition.

## Introduction

**Figure 1:**
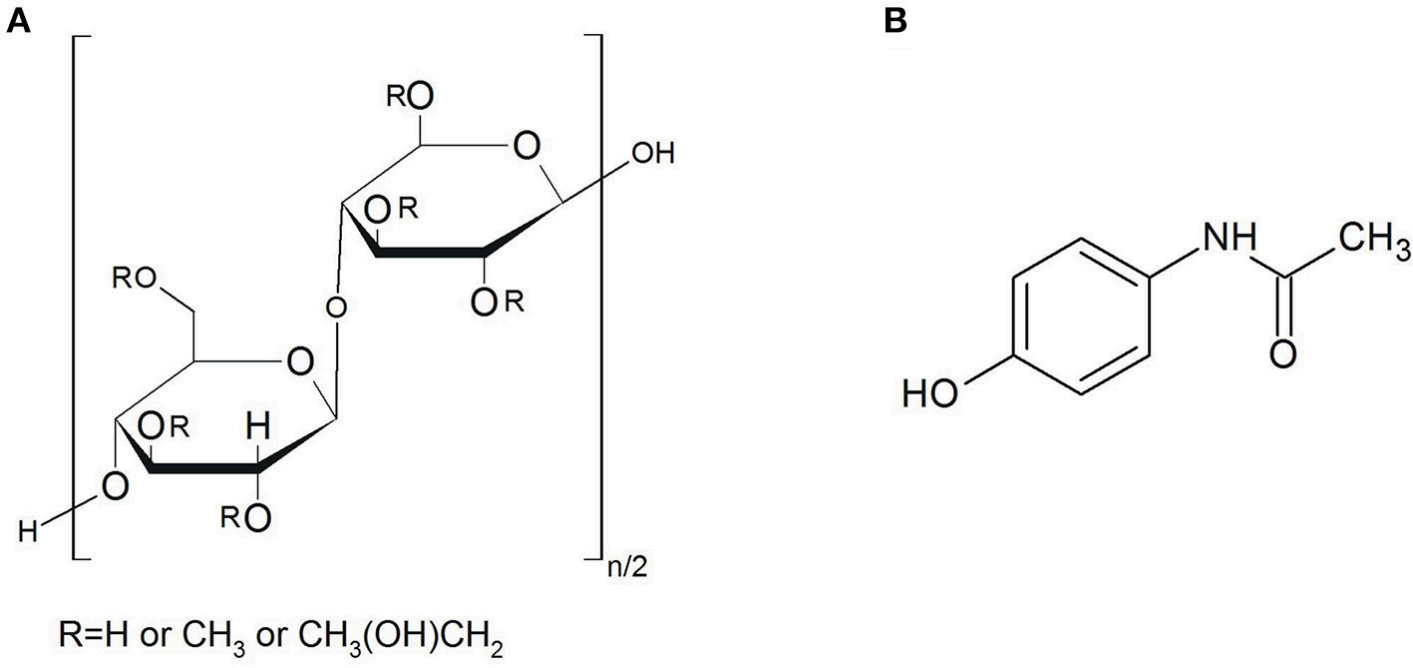
Chemical structure of **(A)** hypromellose and **(B)** paracetamol.

Hydroxypropyl methylcellulose (hypromellose, HPMC, MHPC) is a widely known excipient used in the pharmaceutical industry for preparing oral, ophthalmic, nasal and topical formulations (Rowe et al., 2009). As shown in Figure 1A, hypromellose is not chemically well-defined, described rather as a partly O-methylated and O-(2-hydroxypropylated) cellulose. The –OH groups of polymer can undergo interaction with –COOH groups of active pharmaceutical ingredient (API) leading to hydrogen bonding, which is more stable than those formed between –OH groups of water and polymer (Yao et al., 2011). For commercial purposes, hypromellose is available in several grades that vary in viscosity and extent of substitution. As a non-toxic, biodegradable and hydrophilic polymer, hypromellose has recently been used for the development of new formulations such as the sustained-release mucosa adhesive, controlled-release pellets, microcapsules and variety of matrix, multilayers and coating sustained-release and controlled-release formulations (Rowe et al., 2009). It is also characterized by high swellability, which significantly effects the release kinetics of incorporated API (Siepmann and Peppas, 2001). Upon contact with water or biological fluid, the API diffuses into the device, resulting in polymer chain relaxation with volume expansion. Subsequently, the incorporated API diffuses out of the system.

The literature data has revealed that hypromellose can interact with some APIs, e.g., hypromellose forms a complex with ofloxacin (Subhashree et al., 2011). Fourier transform infrared

(FT-IR) and Raman spectroscopic investigations suggested chemical interaction in formulation leading to hydrogen bonding and esterification. Powder X-ray diffraction (PXRD) and scanning electron microscopy (SEM) data revealed that the crystalline nature of ofloxacin in formulation is stable and would lead to increased stability and API loading, decreasing solubility and delaying release of ofloxacin from polymeric suspension with improved bioavailability and penetration capacity. The study on sustained-release tablets and water solutions with carbamazepine revealed that hypromellose inhibits the transformation of API to its dihydrate in the gel layer of hydrated tablets and water solutions and induces amorphization of API crystals (Katzhendler et al., 1998). This may be due to hydrogen bonding, the –OH groups of polymer possibly attaching to carbamazepine at the site of water binding. Thus, transformation of API to dihydrate is inhibited. The study on nicotinamide mixtures with hypromellose using differential scanning calorimetry (DSC) and PXRD analyses showed that the polymer dissolves in fused API at 140°C (Hino and Ford, 2001). It leads to a decrease in crystallinity and an increase in glass transition temperature of the cooled mixtures as the weight fraction of polymer increased. Dissolution of hypromellose in molten API was accompanied by hydrogen bonding confirmed by FT-IR. The FT-IR, XRDP, and DSC methods were also used to explain the mechanism of formation of amorphous white film on tablets, when the coating solution included hypromellose and calcium lactate pentahydrate (Sakata et al., 2006). The results confirmed the interaction of polymer atoms with ionic calcium.

A brief specification of hypromellose interactions with APIs proved that the polymer helps to reduce the crystallinity of API depending on concentration (Katzhendler et al., 1998; Friedrich et al., 2005; Bajdik et al., 2008; Bhise and Rajkumar, 2008; Zaini et al., 2014; Oh et al., 2015; Chonkar et al., 2016), that it dissolves in fused API (Hino and Ford, 2001) or that APIs dissolve in the polymer (Tian et al., 2015; Baghel et al., 2016), and that it is able to form stable dispersion with no tendency to aggregation (miscibility with APIs) (Marsac et al., 2006; Meng et al., 2015; Tian et al., 2015; Baghel et al., 2016). The formation of hydrogen bonding with APIs is also suggested in many studies (Katzhendler et al., 1998; Subhashree et al., 2011). It should be emphasized that hypromellose contains a large number of hydrogen bonding groups, hence its strong influence over the modification of the crystal habit (Raghavan et al., 2001). Taking all the above into consideration, the purpose of this study was to investigate the effect of hypromellose on the solubility, miscibility and amorphization of paracetamol in mixture with this polymer. Therefore, homogenized mixtures of paracetamol with hypromellose were studied using DSC, hot-stage microscopy (HSM), FT-IR and Raman methods to obtain a fuller understanding of the interactions between ingredients in solid state, including phase diagram construction for crystalline API and amorphous polymer. As far as the authors are aware, this is one of the first studies in which the interactions between paracetamol and hypromellose in the solid state have been evaluated through the methodology outlined below.

Paracetamol (as marketed in Europe, acetaminophen in the USA and Asia), was chosen for the study because it is the most widely used over-the-counter analgesic (pain reliever) and antipyretic (fever reducer) (Bennett and Brown, 2008). Chemically, it is *N*-(4-hydroxyphenyl) acetamide, which formula is presented in Figure 1B. A study on the structure of molecule and crystal of paracetamol revealed that hydrogen bonds only exist among molecules, i.e., API crystal consists of the –OH···O = C and –NH···OH hydrogen bonding (An et al., 2008). The majority of paracetamol formulations are available on the pharmaceutical market in solid dosage forms such as classical, coated and uncoated tablets, tablets with prolonged action, capsules containing powders, pellets or granulates with prolonged release, dragées, and suppositories. There are also liquid dosage forms such as ophthalmic preparations and suspensions for children. Paracetamol in solid dosage forms is used both in single-ingredient formulations, and in combination with opioids (codeine, dihydrocodeine, oxycodone, tramadol), non-steroid anti-inflammatory drugs (ibuprofen), psycholeptics (WHO *Collaborating Centre for Drug Statistics Methodology*, 2018) and antispasmodic drug (eperisone hydrochloride) (Locatelli et al., 2015). Hypromellose is one of the main excipients commonly used for preparation of solid formulations with paracetamol. Recently, extensive studies have been performed on solid-state characterization of paracetamol metastable polymorphic form formed in binary mixtures with hypromellose (Rossi et al., 2003) and thermal behavior of paracetamol in binary mixtures with this polymer (Giordano et al., 2002). It has been revealed that only amorphous paracetamol is present in hypromellose mixtures with API content below 75%, after the cooling phase. Moreover, review papers have also been published on thermal analyses of hypromellose powder, gels and matrix tablets (Ford and Mitchell, 1995; Ford, 1999).

## Materials and Methods

### Materials

Hypromellose (purity ≥ 99%) and paracetamol (purity ≥ 99%) were obtained from Sigma-Aldrich (Steinheim, Germany). Both hypromellose and paracetamol were used in this study as received.

Binary physical mixtures of API and excipient consisting of 5, 10, 20, 30, 40, 50, 60, 70, 80, 90, and 95% of paracetamol were prepared by a gentle mixing of ingredients in a porcelain mortar using a plastic spatula and a pestle. An analytical balance model XA 105 Dual Range (Mettler Toledo, Schwerzenbach, Switzerland), was used to weigh the paracetamol and hypromellose. To achieve complete homogenization, API and excipient were thoroughly mixed over a period of 10 min.

### Methods

DSC curves were recorded on a heat-flux DSC 822^e^ instrument (Mettler Toledo, Schwerzenbach, Switzerland), with a liquid nitrogen cooling system (Dewar vessel) and STAR^e^ 9.10 software. The samples, ~ 4 mg in weight, were accurately weighed (±0.01 mg) and placed in 40 μl flat-bottomed aluminum pans with crimped-on lids. Measurements in triplicate were performed over the temperature range of 25 to 300°C at a heating rate 10°C/min under nitrogen stream (purity 99.9997%, Air Products, Warsaw, Poland) at a flux rate of 70 ml/min.

A heating-cooling-heating test was carried out using the following temperature program: heating samples at a rate of 10°C/min from 25 to 250°C, isotherm for 2 min, cooling at a rate 40°C/min from 250 to −25°C, isotherm for 2 min and the second heating at a rate 10°C/min to 250°C.

Indium (In, purity 99.999%) and zinc (Zn, purity 99.998%) standards (Mettler Toledo, Schwerzenbach, Switzerland) were used to calibrate a DSC cell. Reference values for onset temperature and heat flow were as follows: 156.6°C and 28.45 J/g (In); 419.6°C and 107.5 J/g (Zn), and for those measured: 156.6°C and 28.80 J/g (In); 420.1°C and 110.7 J/g (Zn).

A HSM equipped with a BX41polarizing microscope (Olympus, Shinjuku, Japan) and a color video digital camera SC30 supported by Olympus CellA software was used to record imagines during temperature scans. A 1–5 mg of sample was placed between two glass cover slides and put on a hot stage (Semic, Bioelektronika, Krakow, Poland) equipped with an SR90 temperature regulator (Shimaden, Tokio, Japan) and Heating Desk Shimaden software. Measurements were performed over the temperature range of 25 to 300°C at a heating rate of 10°C/min.

FT-IR spectra were collected using a Nicolet 380 FT-IR spectrometer (Thermo Fischer Scientific, Madison, USA), with a DTGS KBr detector and OMNIC software. The samples analyzed were prepared as KBr pellets with the aid of a hydraulic press (Specac, Orpington, UK), each pellet being prepared from a 1-mg sample and 100 mg of spectroscopy-grade KBr (Merck, Darmstadt, Germany). Measurements in triplicate were performed in the 4,000–400 cm^−1^ spectral region with a spectral resolution of 4 cm^−1^. Prior to each measurement, background spectra was taken with an average of 16 scans.

Raman spectra were recorded on a DXR SmartRaman spectrometer (Thermo Fisher Scientific, Madison, USA). The instrument was equipped with a Raleigh filter, CCD detector and OMNIC software. Measurements were run in triplicate over a spectral range of 3413–99 cm^−1^ with resolution of 2 cm^−1^. A 15-mW DXR 780 nm laser with an aperture of 25 μm was deployed. Exposure time was 1 s (twice).

### Calculations

The solubility curve of crystalline API in an amorphous polymer was calculated in accordance with the procedure described in the literature (Tian et al., 2015; Baghel et al., 2016). If a polymer is considered as a solvent, the quantity of paracetamol soluble in hypromellose is expressed as the mole fraction (*x*_*API*_) of the dissolved API in relation to the activity coefficient (γ_*API*_), according to the equation:

(1) $$\ln x_{API}=\frac{\Delta H_{fus}}{RT_{m}}\left(1-\frac{Tm}{T}\right)-\ln\gamma_{API}$$

where: Δ*H*_*fus*_ is the melting enthalpy of paracetamol, *R* the gas constant, *T*_*m*_ the melting temperature of paracetamol in Kelvin, and *T* the temperature in Kelvin of the two phases of paracetamol in equilibrium.

The activity coefficient (γ_*API*_) of paracetamol can be calculated on the basis of Hansen solubility parameters (δ), the molar volume (*V*) of mixture ingredients (*k*), the mixture volume ($\bar{V}$) and the molar volume of weighted Hansen solubility parameter ($\bar{\delta }$), according to the equation:

(2) $$\begin{array}{rcl} \ln\gamma_{API} & = & \frac{V_{API}}{RT}\left\{{\left(\delta_{d}^{API}-{\bar{\delta }}_{d}\right)}^{2}+0,25\left[{\left(\delta_{p}^{API}-{\bar{\delta }}_{p}\right)}^{2}+{\left(\delta_{h}^{API}-{\bar{\delta }}_{h}\right)}^{2}\right]\right\} \end{array}$$

The mixture volume ($\bar{V}$) and molar volume of weighted Hansen solubility parameter ($\bar{\delta }$), taking into account the character of interactions, i.e., dispersion forces (δ_*d*_), polar interactions (δ_*p*_) and hydrogen bonding (δ_*h*_), can be calculated according to the following equations:

(3) $$\begin{array}{l} \bar{\delta }=\sum_{k=1}^{n}\phi_{k}\delta_{k}\text{for}{\bar{\delta }}_{d},\;{\bar{\delta }}_{p},\;{\bar{\delta }}_{h},\text{where}k\text{istheparacetamol}\\ \;orhypromellose \end{array}$$

(4) $$\phi_{k}=\frac{x_{k}V_{k}}{\bar{V}}$$

(5) $$\bar{V}=\sum_{k=1}^{n}x_{k}V_{k}$$

(6) $$V_{k}=\frac{M_{k}}{\rho_{k}}$$

where ϕ is the volume fraction of API or polymer (*k*), *x* the mole fraction, *M* the molecular weight, and ρ the density of ingredients.

The miscibility curve of paracetamol with hypromellose can be predicted based on the Florry-Huggins theory and the Gibbs free energy (Δ*G*_*mix*_) (Forster et al., 2001; Djuris et al., 2013; Maniruzzaman et al., 2014; Tian et al., 2015). The Gibbs free energy (Δ*G*_*mix*_) of mixing both ingredients is described by the equation:

(7) $$\Delta G_{mix}=RT\left[\phi_{API}\ln\phi_{API}+\frac{1-\phi_{API}}{n}\ln\left(1-\phi_{API}\right)+\chi \phi_{API}\left(1-\phi_{API}\right)\right]$$

where *n* is the number of API lattice sites, defined as the volume of API molecule occupied by a polymer chain, and χ is the API-polymer interaction parameter.

The number of API lattice sites occupied by a polymer chain (*n*) and the API-polymer interaction parameter (χ) can be calculated by the following equations:

(8) $$n=\frac{V_{polymer}}{V_{API}}$$

(9) $$\chi =\frac{v{\left(\delta_{API}-\delta_{polymer}\right)}^{2}}{RT}$$

(10) $$\delta_{k}=\;\sqrt{\delta_{d}^{2}+\delta_{p}^{2}+\delta_{h}^{2}}$$

where *v* is the volume of paracetamol lattice site (volume of the API), and δ is the Hansen solubility parameter (δ).

Miscibility curve (spinodal curve) can be predicted by setting the second derivative of the Gibbs free energy to zero, as expressed by the equation:

(11) $$T_{s}=\frac{2v{\left(\delta_{API}-\delta_{polymer}\right)}^{2}}{R}*\frac{1}{\frac{1}{\phi_{API}}-\frac{1}{m(1-\phi_{API})}}$$

The glass transition curve can be predicted using the relation between the glass transition temperature (*T*_*g*_) of the mixture and the weight fraction of paracetamol (*w*_*API*_) in the mixture, according to the Fox equation (Tian et al., 2015):

(12) $$\frac{1}{T_{g}}=\frac{w_{API}}{T_{g_{API}}}+\frac{1-w_{API}}{T_{g_{polymer}}}$$

where *T*_*g*_ is the glass transition temperature of a mixture, *T*_*g*_*API*__ and *T*_*g*_*polymer*__ are the glass transition temperatures of paracetamol and hypromellose, respectively.

## Results and Discussion

Paracetamol may interact with other APIs or excipients when used together, e.g., many drug formulations including paracetamol and caffeine irrespective of the fact that both active ingredients form a eutectic mixture (Bi et al., 2003). Because hypromellose is commonly used as an important excipient in the fabrication of paracetamol drug formulations, the current study was carried out to assess potential interactions between ingredients mixed at differing mass ratios.

### DSC of Paracetamol-Hypromellose Mixtures

**Figure 2:**
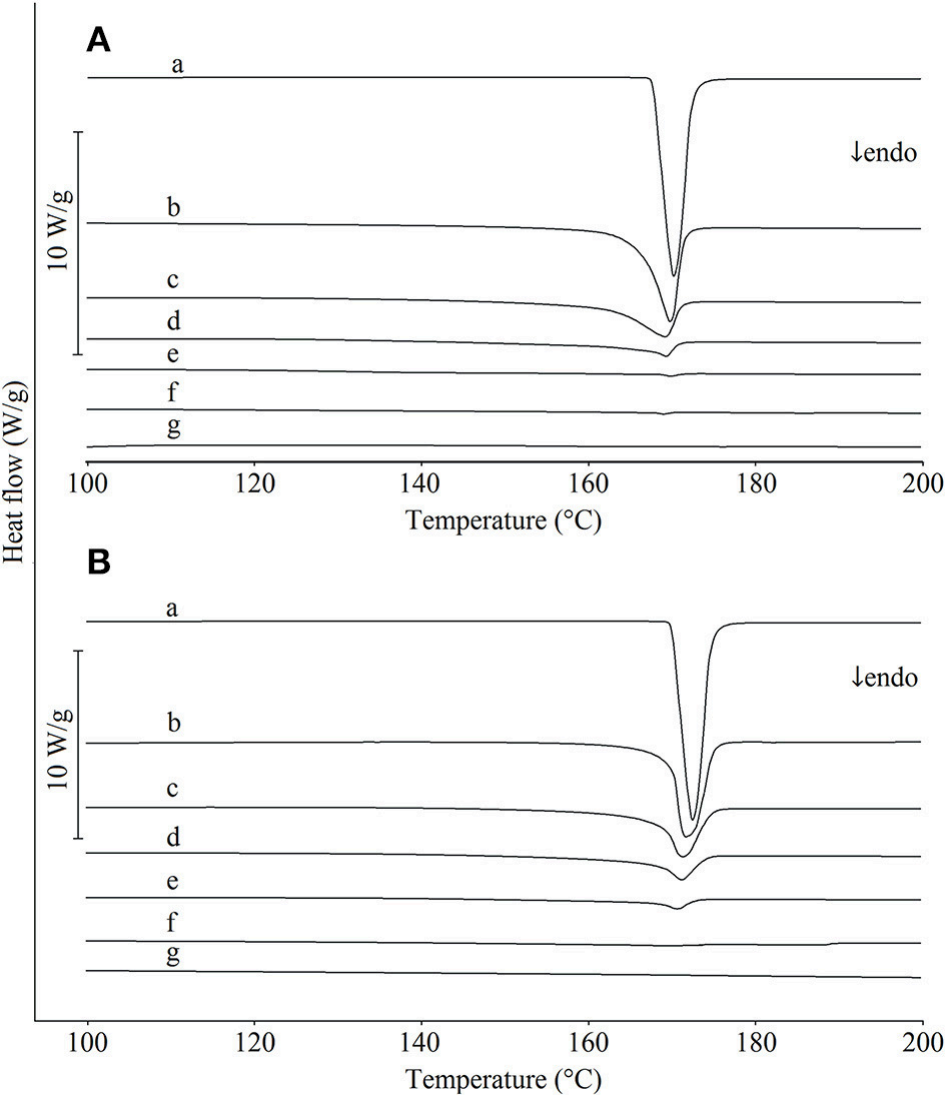
DSC curves for (a) paracetamol, (g) hypromellose and their mixtures containing (b) 80%, (c) 60%, (d) 40%, (e) 20%, and (f) 10% of paracetamol. Mixtures mixed with **(A)** a plastic spatula and **(B)** a pestle.

The DSC curves of paracetamol, hypromellose and their mixtures prepared by carefully mixing ingredients with a plastic spatula in a porcelain mortar are shown in Figure 2A, those mixed using a pestle in Figure 2B. The DSC curve of paracetamol (curve a) displays a single endothermic peak due to the melting of polymorphic form I (monoclinic) at 169.4°C, which is consistent with the literature data (Klìmová and Leitner, 2012). The physical mixtures homogenized using a spatula (Figure 2A, curves b-f) revealed that the endothermic peak due to the melting of paracetamol appeared on the DSC curves over the entire concentration range and decreased as the content of active ingredient in the mixture decreased. By contrast, DSC curves for mixtures prepared using a pestle (Figure 2B, curves b-f) did not display the melting peak of paracetamol for mixtures containing < 20% of API. This suggests that the interaction between paracetamol and hypromellose resulted in a partial reduction in paracetamol crystallinity, probably due to intermolecular interaction with the formation of hydrogen bonding. As reported in the literature, other physical interactions are also possible, e.g., formation of eutectic mixtures (Bi et al., 2003), solid dispersions (Jahangiri et al., 2015), dissolution of paracetamol in hypromellose (Tian et al., 2015), reduction of paracetamol crystallinity in the presence of microcrystalline cellulose (de Oliveira et al., 2017) and amorphization or polymorphic transformation (Salunkhe et al., 2018). The risk of initiating interactions between ingredients is greater when homogenization of the mixtures is carried out using a pestle (Byard et al., 2005; Curtin et al., 2013).

The data compiled in Table 1 shows that the correlation coefficients between the enthalpy of paracetamol melting and the API content in mixtures with hypromellose are high. Detailed inspection of other regression parameters revealed high values of intercept (*b*) in calibration equations for mixtures mixed using either plastic spatula or pestle. However, the intercept value increased approximately 3-fold for mixtures mixed with a pestle as opposed to those mixed with a spatula. This may be indicative of the fact that the heat of fusion values are lower than those expected for real content of paracetamol in the mixtures. The implication is that the immediate cause of this situation may be a reduction in crystallinity of paracetamol due to its contact with hypromellose. The intensity of this process is enhanced by mixing the ingredients with a pestle.

**Table 1:** Regression parameters of calibration curves and limits of detection and determination of paracetamol methods in mixtures with hypromellose.

**Parameter**	**Hypromellose**	
	**Mixing with a spatula**	**Mixing with a pestle**
Paracetamol content (%)	5–95	5–95
a ±Δa	1.85 ± 0.09	1.81 ± 0.17
S_a_	0.02	0.04
b ±Δb	−12.39 ± 5.81	−37.45 ± 10.16
S_b_	1.35	2.36
r	0.9986	0.9986
S_xy_	2.83	2.74

### Hot-Stage Microscopy

To investigate potential interactions between ingredients, HSM was also used. This technique is a combination of microscopy and thermal analysis to enable the investigation of solid state changes in substances of pharmaceutical interest under heating or cooling (Steger et al., 2012). It allows changes in appearance of paracetamol crystals, particles of amorphous hypromellose and their mixtures to be observed, along with the determination of characteristic temperatures.

**Figure 3:**
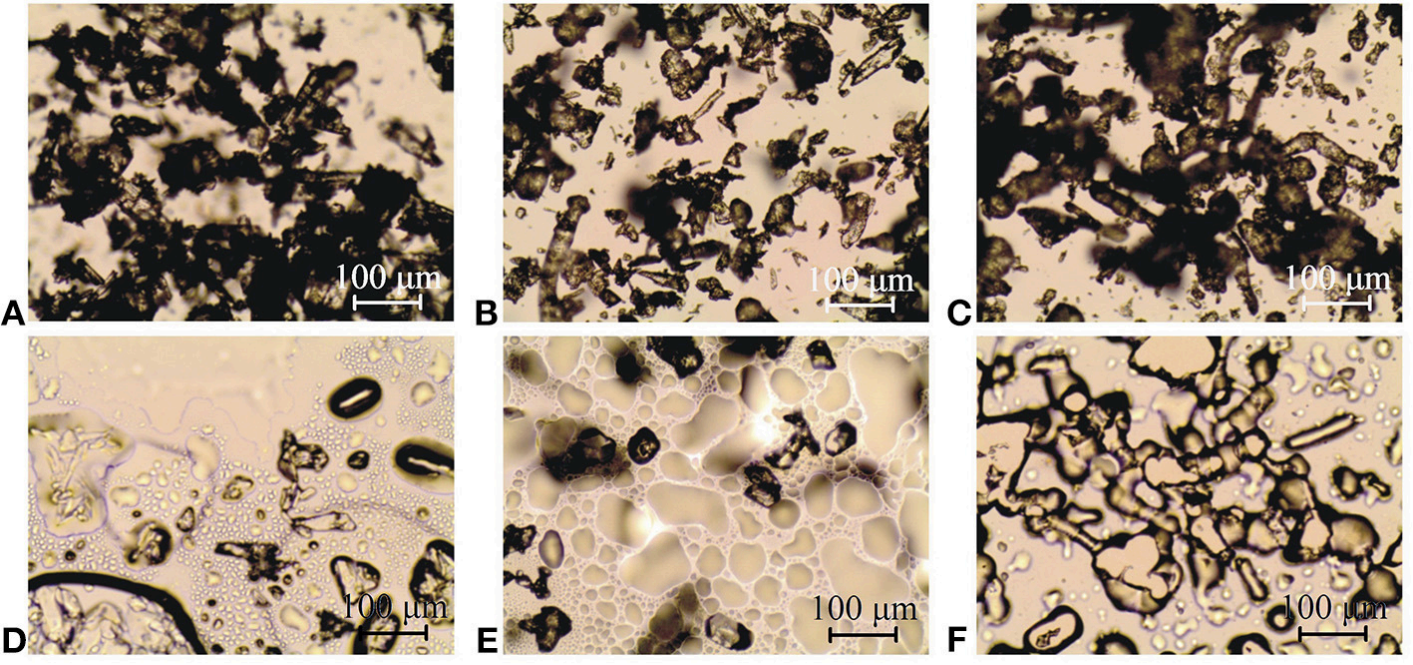
HSM images of **(A,D)** paracetamol, **(C,F)** hypromellose and **(B,E)** their mixture at 1:1 mass ratio at ambient temperature **(A–C)**, after melting of paracetamol **(D,E)** and after liquidation of hypromellose **(F)**.

The appearance of paracetamol, hypromellose and their mixture at a 1:1 mass ratio is shown in Figures 3A–C, respectively (ambient temperature), and Figures 3D,E (after melting of paracetamol) and Figure 3F (after liquidation of hypromellose). Image a reflects the fact that between ambient and sublimation temperature API crystals were characterized by regular shape but various sizes. Under heating, paracetamol sublimed above 150°C, and subsequently, its small crystals melted at ~ 168°C. Vapors of API sublimation solidified to form needle-like crystals on the cool surface. FT-IR used to investigate these crystals confirmed the existence of form I of paracetamol. DSC analyses revealed that the needle-like crystals melted at 168°C, which corroborates the melting of form I of paracetamol (Klìmová and Leitner, 2012). Complete melting of API and its intensive evaporation were observed above 172°C (image d). Under cooling to ambient temperature, the sample crystallized. On the other hand, image c presents particles of amorphous hypromellose at various sizes. Under heating, liquidation and browning of the polymer were observed at ~ 260°C (Figure 3F).

A HSM study revealed that at the initial stage of the heating process, API crystals and particles of amorphous polymer were easily visible (Figure 3B) in mixtures containing 10, 30, 50, 70, and 90% of paracetamol. Next, as with paracetamol heating, the sublimation of mixtures commenced at ~ 150°C. Afterwards, the melting of the finest crystals began at temperatures lower than that of paracetamol, i.e., ~ 162, 161, 159, 157, and 156°C, respectively, for mixtures with decreasing content of API. Figure 3E presents the 1:1 mass ratio mixture heated to 159°C and shows hypromellose particles and melted paracetamol. In brief, neither new crystals nor amorphous particles were found when the mixtures were heated.

### FT-IR and Raman Spectroscopy

**Figure 4:**
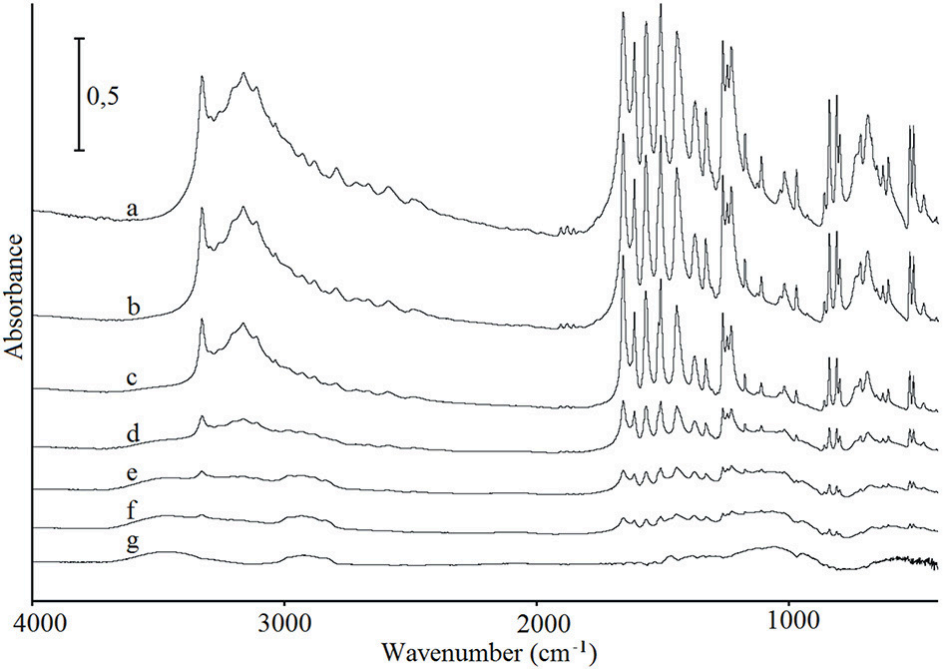
FT-IR spectra for (a) paracetamol, (g) hypromellose and their mixtures containing (b) 80%, (c) 60%, (d) 40%, (e) 20%, and (f) 10% of paracetamol.

FT-IR and Raman spectroscopy were used to investigate whether any intermolecular interactions or chemical reactions may have occurred between paracetamol and hypromellose. Figure 4 shows the FT-IR spectra of paracetamol, hypromellose, and their mixtures. In interpreting these data, special attention was paid to the characteristic bands of chemical groups capable of forming hydrogen bonding. Characteristic bands for monoclinic paracetamol (spectrum a) were found at 808 cm^−1^ (amid group deformation), 968 and 1,259 cm^−1^ (C–N stretching vibrations), 1,655 cm^−1^ (C = O stretching and C–NH deformation), 3,034 cm^−1^ (N–H stretching) and 3,161 and 3,325 cm^−1^ (O–H stretching) (Burgina et al., 2004; An et al., 2008). This confirms the results of DSC analysis which indicates that form I of paracetamol is the API under investigation (Burgina et al., 2004; Łuczak et al., 2013). Detailed inspection of the FT-IR data for mixtures (spectra b–f) revealed that characteristic absorption bands of paracetamol were found in all the spectra. Their intensity decreases proportionate to decreasing paracetamol content in the mixtures. Moreover, the lack of shifting in the characteristic absorption bands of both ingredients in conjunction with a lack of new bands suggests that neither physical interaction nor chemical reaction occurred between ingredients. It concurs with the literature data which revealed that hydrogen bonds have not been formed between ingredients in the physical mixtures, but they can be formed during hot-melt extrusion, e.g., nimodipine with hydroxypropyl methylcellulose succinate acetate (Zhang et al., 2018). It has also been confirmed that shifting of NH band at 3,300 cm^−1^ to 3,357 cm^−1^ in the spectrum of nimodipine extrudate with polymer strongly evidenced the formation of hydrogen bonds between the amine and carbonyl groups of API and polymer, respectively. The hydrogen bonds can also be created in the presence of solvent, e.g., using aqueous polymer solutions (Wen et al., 2005) or during electrospray-drying (Liu et al., 2018).

**Figure 5:**
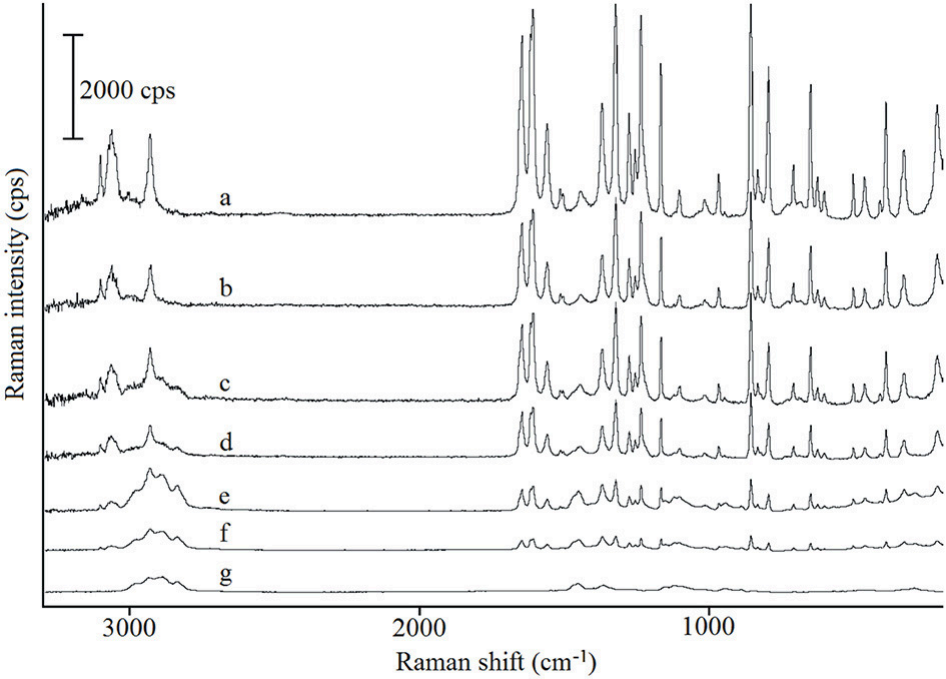
Raman spectra for (a) paracetamol, (g) hypromellose and their mixtures containing (b) 80%, (c) 60%, (d) 40%, (e) 20%, and (f) 10% of paracetamol. Raman spectra were obtained using laser at 780 nm wavelength.

The Raman spectra of paracetamol, hypromellose and their mixtures are shown in Figure 5. Characteristic bands for paracetamol (spectrum a) were found at 463 cm^−1^ (out-of-plane ring deformation), 1,235 cm^−1^(C–N stretching vibration), 1,560 cm^−1^(N–H and C = O stretching), and 1,646 cm^−1^ (C = O stretching) (Burgina et al., 2004). Raman shift at 463 cm^−1^ is particularly characteristic of the polymorphic form I of paracetamol in contrast to that at 454 cm^−1^ which is characteristic of form II (An et al., 2008). Thus, similar to the DSC and FT-IR data, form I of paracetamol has also been identified by Raman spectroscopy. Detailed inspection of the data for all API mixtures (spectra b-f) confirmed that the existence of characteristic Raman shifts is related to paracetamol. The intensity of these bands decreases in line with decreased paracetamol content in the mixtures. Under no circumstances did bands shift or new bands appear. Therefore, neither physical interactions nor chemical reactions between ingredients were to be expected.

### Phase Diagram for Paracetamol and Hypromellose

The classical phase diagram is constructed using the melting temperatures of two ingredients existing in a crystal form. Hypromellose is an amorphous polymer that does not melt, but is characterized by glass transition. Preparation of the phase diagram for a mixture of crystalline API and amorphous polymer entails the calculation of three curves which predict the solubility of API in polymer, mutual miscibility of ingredients and temperature of glass transition. The phase diagram reflects an equilibrium state predicted from the calculations and enables the conclusions to be drawn relating to the quantity of API (that may be dissolved in polymer at a given temperature) and about the composition of the mixture characterized by complete miscibility at a given temperature.

**Figure 6:**
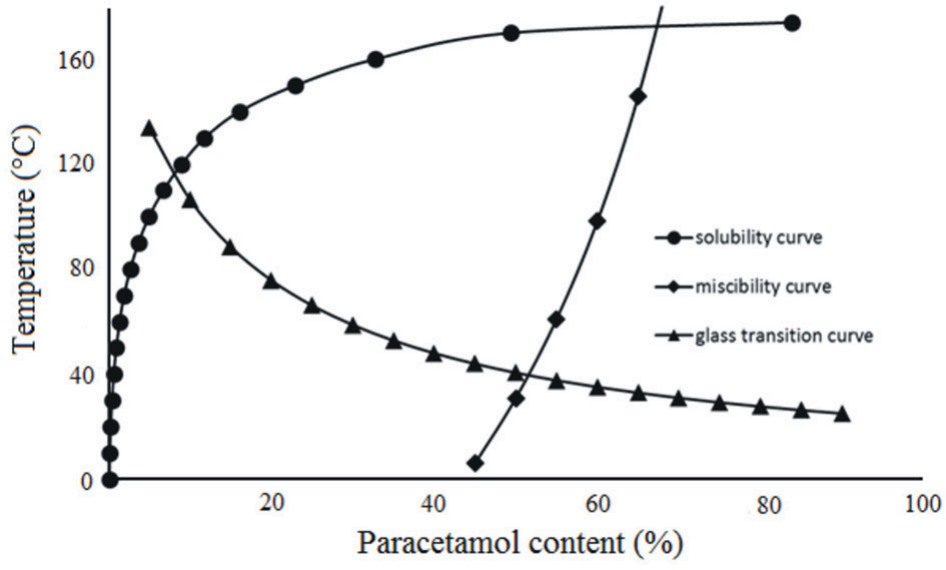
Phase diagram for paracetamol binary mixtures with hypromellose.

The literature data used for calculations of the solubility, miscibility, and glass transition curves in equations 1, 11, and 12 (Calculations section) are listed in Table 2 (Espeau et al., 2005; Chan et al., 2006; Sibik et al., 2014; Adhikari et al., 2015), while the calculated values predicting the solubility of paracetamol in polymer, mutual miscibility of ingredients and the glass transition of mixture are compiled in Tables 3, 4. These data were used to prepare the phase diagram for physical mixture of API with hypromellose shown in Figure 6.

**Table 2:** Parameters used to develop a phase diagram.

**Parameter**	**Hypromellose**	**Paracetamol**
Molar mass (g/mol)	10,000	151.16 (Espeau et al., 2005)
Density (g/cm^3^)	1.0 (Chan et al., 2006)	1.3 (Espeau et al., 2005)
Enthalpy of melting (J/mol)	–	29716
Molar volume (cm^3^/mol)	–	139.0 (Chan et al., 2006)
Volume of a lattice (Å^3^)	–	772.26 (Adhikari et al., 2015)
Glass transition (K)	433.15	296 (Djuris et al., 2013)
Melting (K)	–	447.9 (Sibik et al., 2014)
**SOLUBILITY PARAMETERS (MPa)**^**1/2**^		
δ_d_	16.95 (Chan et al., 2006)	20.74 (Chan et al., 2006)
δ_p_	8.55 (Chan et al., 2006)	12.7 (Chan et al., 2006)
δ_h_	9.04 (Chan et al., 2006)	17.49 (Chan et al., 2006)
δ	21.03	29.96

*References are given in the parentheses*.

**Table 3:** Calculated quantities of paracetamol that can be dissolved in hypromellose in relation to the temperature.

**Quantity of paracetamol (%)**	**Temperature (°C)**																
	**0**	**10**	**20**	**30**	**40**	**50**	**60**	**70**	**80**	**90**	**100**	**110**	**120**	**130**	**140**	**150**	**160**
95	0.75	1.20	1.84	2.75	4.01	5.71	7.97	10.89	14.63	19.34	25.18	32.34	41.01	51.39	63.71	78.17	95.02
90	0.71	1.13	1.74	2.60	3.79	5.40	7.53	10.30	13.84	18.29	23.83	30.61	38.82	48.65	60.32	74.03	
85	0.67	1.06	1.63	2.44	3.56	5.08	7.09	9.70	13.04	17.24	22.46	28.86	36.62	45.91	56.94	69.90	
80	0.62	0.99	1.53	2.29	3.34	4.76	6.64	9.10	12.24	16.19	21.10	27.13	34.44	43.20	53.59	65.82	
75	0.58	0.92	1.42	2.13	3.11	4.44	6.21	8.51	11.45	15.16	19.77	25.43	32.30	40.54	50.32	61.82	
70	0.54	0.85	1.32	1.98	2.90	4.14	5.78	7.93	10.68	14.15	18.48	23.78	30.22	37.95	47.13	57.94	
65	0.49	0.79	1.22	1.83	2.68	3.84	5.37	7.37	9.94	13.18	17.22	22.18	28.21	35.45	44.06	54.19	
60	0.45	0.73	1.12	1.69	2.48	3.55	4.98	6.84	9.23	12.25	16.02	20.65	26.28	33.05	41.10	50.60	
55	0.42	0.67	1.03	1.56	2.29	3.28	4.60	6.33	8.55	11.36	14.87	19.18	24.44	30.76	38.28	47.16	
50	0.38	0.61	0.95	1.43	2.10	3.02	4.24	5.84	7.90	10.51	13.77	17.79	22.69	28.58	35.60	43.90	
45	0.35	0.56	0.87	1.31	1.93	2.78	3.90	5.39	7.29	9.71	12.74	16.48	21.03	26.52	33.07	40.80	
40	0.31	0.51	0.79	1.20	1.77	2.55	3.59	4.96	6.72	8.96	11.77	15.24	19.47	24.58	30.67	37.89	
35	0.29	0.46	0.72	1.09	1.62	2.33	3.29	4.55	6.18	8.26	10.86	14.07	18.00	22.75	28.42		
30	0.26	0.42	0.66	1.00	1.48	2.13	3.02	4.18	5.68	7.60	10.00	12.98	16.63	21.04	26.31		
25	0.23	0.38	0.60	0.91	1.35	1.95	2.76	3.83	5.22	6.99	9.21	11.97	15.35	19.44	24.34		
20	0.21	0.34	0.54	0.82	1.23	1.78	2.52	3.51	4.78	6.42	8.47	11.02	14.15	17.94			
19	0.21	0.34	0.53	0.81	1.20	1.75	2.48	3.44	4.70	6.31	8.33	10.84	13.92	17.66			
18	0.20	0.33	0.52	0.79	1.18	1.71	2.43	3.38	4.62	6.20	8.19	10.66	13.70	17.38			
17	0.20	0.32	0.51	0.78	1.16	1.68	2.39	3.32	4.54	6.09	8.05	10.49	13.47				
16	0.19	0.32	0.50	0.76	1.14	1.65	2.35	3.27	4.46	5.99	7.92	10.31	13.26				
15	0.19	0.31	0.49	0.75	1.12	1.62	2.30	3.21	4.38	5.89	7.78	10.14	13.04				
14	0.19	0.30	0.48	0.73	1.09	1.59	2.26	3.15	4.31	5.79	7.65	9.98	12.83				
13	0.18	0.30	0.47	0.72	1.07	1.56	2.22	3.09	4.23	5.69	7.52	9.81	12.62				
12	0.18	0.29	0.46	0.71	1.05	1.53	2.18	3.04	4.16	5.59	7.40	9.65					
11	0.17	0.29	0.45	0.69	1.03	1.50	2.14	2.99	4.08	5.49	7.27	9.49					
10	0.17	0.28	0.44	0.68	1.01	1.48	2.10	2.93	4.01	5.40	7.15	9.33					
9	0.17	0.27	0.43	0.67	0.99	1.45	2.06	2.88	3.94	5.31	7.03						
8	0.16	0.27	0.42	0.65	0.98	1.42	2.03	2.83	3.87	5.21	6.91						
7	0.16	0.26	0.42	0.64	0.96	1.39	1.99	2.78	3.81	5.12							
6	0.16	0.26	0.41	0.63	0.94	1.37	1.95	2.73	3.74	5.04							
5	0.15	0.25	0.40	0.62	0.92	1.34	1.92	2.68	3.67	4.95							
4	0.15	0.25	0.39	0.60	0.90	1.32	1.88	2.63	3.61								
3	0.15	0.24	0.38	0.59	0.89	1.29	1.85	2.58									
2	0.14	0.24	0.38	0.58	0.87	1.27	1.81										

**Table 4:** Calculated temperatures of miscibility and glass transition in the mixtures of paracetamol with hypromellose.

**Content of paracetamol (%)**	**Mole fraction (*****x*****)**		**Volume fraction (Φ)**		**Miscibility temperature (°C)**	**Glass transition temperature (°C)**	
	**Paracetamol**	**Hypromellose**	**Paracetamol**	**Hypromellose**		**Calculated**	**DSC**
95	0.9992	0.0008	0.938	0.062	2097.6	23.9	
90	0.9983	0.0017	0.877	0.123	1029.3	25.0	24.13
85	0.9973	0.0027	0.818	0.182	631.2	26.3	
80	0.9962	0.0038	0.760	0.240	423.1	27.7	
75	0.9950	0.0050	0.704	0.296	295.1	29.2	
70	0.9936	0.0064	0.649	0.351	208.4	31.0	26.09
65	0.9919	0.0081	0.596	0.404	145.8	32.9	
60	0.9900	0.0100	0.543	0.457	98.3	35.1	
55	0.9878	0.0122	0.492	0.508	61.0	37.6	
50	0.9851	0.0149	0.442	0.558	30.9	40.6	30.50
45	0.9819	0.0181	0.394	0.606	6.0	44.0	
40	0.9778	0.0222	0.346	0.654	−15.0	48.0	
35	0.9727	0.0273	0.299	0.701	−33.1	52.8	
30	0.9659	0.0341	0.254	0.746	−49.1	58.8	35.59
25	0.9566	0.0434	0.209	0.791	−63.6	66.2	
20	0.9430	0.0570	0.166	0.834	−77.1	75.8	
19	0.9395	0.0605	0.157	0.843	−79.8	78.0	
18	0.9356	0.0644	0.148	0.852	−82.5	80.4	
17	0.9313	0.0687	0.140	0.860	−85.2	83.0	
16	0.9265	0.0735	0.131	0.869	−88.0	85.7	
15	0.9211	0.0789	0.123	0.877	−90.8	88.6	
14	0.9150	0.0850	0.114	0.886	−93.7	91.7	
13	0.9081	0.0919	0.106	0.894	−96.6	95.0	
12	0.9002	0.0998	0.098	0.902	−99.8	98.6	
11	0.8910	0.1090	0.089	0.911	−103.0	102.5	
10	0.8802	0.1198	0.081	0.919	−106.5	106.7	60.44
9	0.8674	0.1326	0.073	0.927	−110.4	111.2	
8	0.8519	0.1481	0.065	0.935	−114.6	116.1	
7	0.8328	0.1672	0.056	0.944	−119.4	121.5	
6	0.8085	0.1915	0.048	0.952	−125.1	127.4	
5	0.7769	0.2231	0.040	0.960	−132.0	133.9	112.34
4	0.7338	0.2662	0.032	0.968	−140.8	143.5	
3	0.6717	0.3283	0.024	0.976	−152.8	152.5	
2	0.5745	0.4255	0.016	0.984	−170.8	162.8	

**Figure 7:**
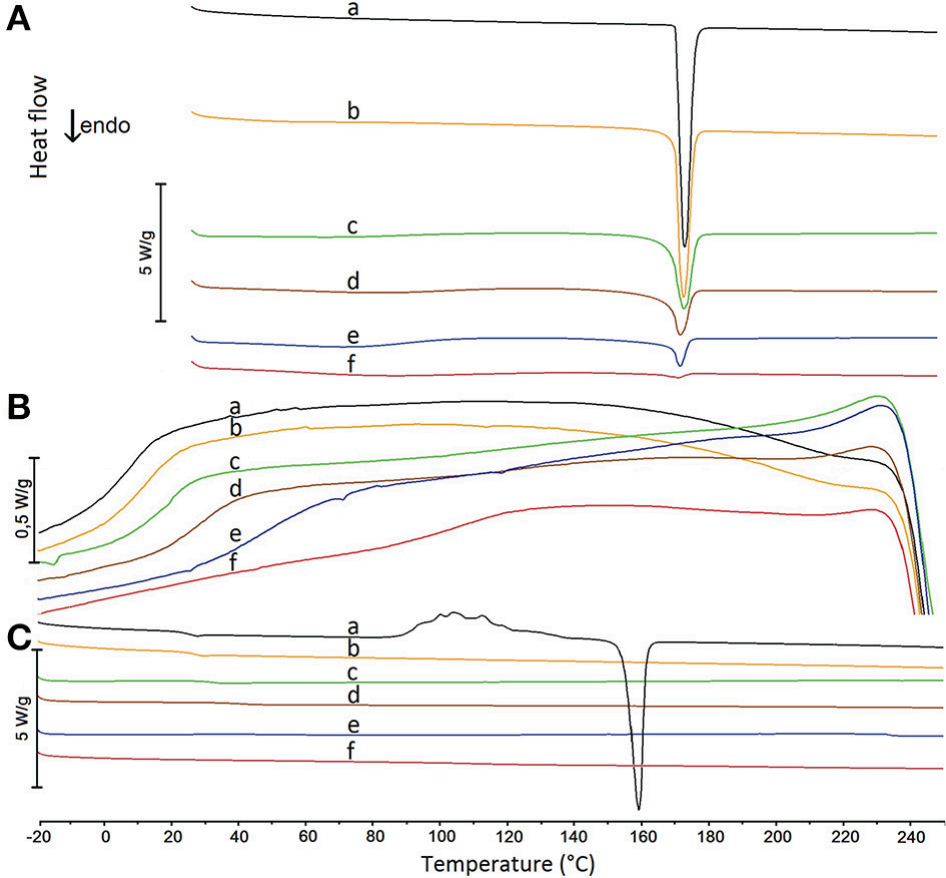
DSC heating and cooling curves for (a) paracetamol and their mixtures containing (b) 90%, (c) 70%, (d) 50%, (e) 30%, and (f) 10% of paracetamol. **(A)** the first heating cycle, **(B)** the cooling cycle, **(C)** the second heating cycle.

The glass transition curve reflects the predicted temperature at which the mixture undergoes glass transition at a given composition. Glass transition was also confirmed with the aid of the heating-cooling-heating test. DSC curves reflecting this test for mixtures containing 90, 70, 50, 30, and 10% of API are illustrated in Figure 7. Detailed inspection of these curves revealed that melted paracetamol underwent amorphization during the fast cooling cycle (Figure 7B, curve a), a fact confirmed by an inflection on the DSC curve due to paracetamol glass transition and the absence of exothermic peak due to the crystallization of API. The performance of all the mixtures under study was the same as paracetamol behavior (Figure 7B, curves b–f).

In the second cycle of heating, the DSC curve of paracetamol (Figure 7C, curve a) showed inflection at ~23°C caused by the glass transition. A further exothermic peak was found due to paracetamol recrystallization to polymorphic form II which melted at ~ 160°C. For all mixtures, the DSC curves of the second heating cycle (Figure 7C, curves b–f) revealed an inflection due to glass transition between ~ 24 and ~ 112°C, respectively, for decreasing quantity of paracetamol in mixture. These temperatures are compiled in Table 4 along with those calculated using a suitable Equation (12). Neither an exothermic peak due to paracetamol crystallization nor an endothermic peak caused by its melting was found on the DSC curves of the second heating cycle. Thus, the melting of paracetamol with hypromellose lead to amorphization of API with inhibition of its crystallization.

The phase diagram permits a prediction of the thermodynamic stability of the system containing API and hypromellose in relation to temperature (Djuris et al., 2013; Tian et al., 2015). Figure 6 shows that the area above the solubility curve is the thermodynamically stable region, while the area between the miscibility and solubility curves represents the thermodynamically unstable region in which spontaneous phase separation may occur. In addition, the area on the right-side of the miscibility curve indicates the region in which ingredients are separated. The glass transition curve represents the glass transition temperatures for paracetamol-hypromellose mixtures.

## Conclusions

DSC studies of paracetamol mixtures with hypromellose revealed potential interaction between ingredients that resulted in a reduction of paracetamol crystallinity. This was confirmed using a heating-cooling-heating cycle—fast cooling of paracetamol mixture with hypromellose caused the amorphization of paracetamol, while crystallization of API was inhibited in the presence of the polymer. There is a greater risk of initiating interactions when the homogenization of mixtures is carried out using a pestle. FT-IR and Raman investigations excluded chemical reaction and hydrogen bonding between API and polymer. Furthermore, HSM research indicated that neither new crystals nor amorphous particles were found when mixtures were heated.

The phase diagram developed for paracetamol mixtures with hypromellose allows us to forecast to what extent API dissolves in the polymer. The phase diagram also reflects the mutual miscibility of ingredients as well as the temperature and composition of mixture at which the system should be physically stable.

## Data Availability Statement

The datasets generated for this study are available from the authors.

## Author Contributions

MW and EL conceived and designed experiments. EL conducted experiments and analyzed data. MW and EL wrote the manuscript. All authors read and approved the manuscript.

### Conflict of Interest Statement

The authors declare that the research was conducted in the absence of any commercial or financial relationships that could be construed as a potential conflict of interest.
